# Clinical efficacy of manipulation under brachial plexus block anesthesia for primary adhesive capsulitis of shoulder: a retrospective cohort study

**DOI:** 10.3389/fsurg.2025.1670743

**Published:** 2025-11-07

**Authors:** Haiyan Zhou, Liming Cheng

**Affiliations:** 1 Department of Orthopaedics, Yueyang Hospital of Integrated Traditional Chinese and Western Medicine, Shanghai University of Traditional Chinese Medicine, Shanghai, China; 2 Department of Orthopaedic Surgery, Tongji Hospital Affiliated to Tongji University, Shanghai, China

**Keywords:** adhesive capsulitis, frozen shoulder, manipulation under anesthesia, brachial plexus block, constant score, range of motion

## Abstract

**Objective:**

To evaluate the clinical efficacy of manipulation under brachial plexus block anesthesia (MUA) compared to standardized conservative treatment in patients with primary adhesive capsulitis of the shoulder (ACS).

**Methods:**

This retrospective cohort study analyzed 72 patients with primary ACS, allocated to either the MUA group (*n* = 36) or the control group receiving conservative treatment (*n* = 36). The MUA group underwent a single manipulation procedure under ultrasound-guided interscalene brachial plexus block, followed by a structured 3-month rehabilitation protocol. The control group received a comprehensive conservative regimen. Primary outcomes included shoulder range of motion (ROM) and Constant-Murley scores, assessed preoperatively and at 1, 3, 6, and 12 months post-intervention.

**Results:**

The MUA group demonstrated significantly greater improvement in all ROM parameters compared to the control group at all follow-up intervals (*P* < 0.001). At 12 months, forward flexion improved to 152.4° ± 8.7° (vs. 101.2° ± 13.5° in controls), abduction to 150.6° ± 10.5° (vs. 95.8° ± 12.3°), and external rotation to 54.6° ± 5.3° (vs. 38.2° ± 5.9°). Constant scores were significantly higher in the MUA group (86.7 ± 3.9 vs. 73.5 ± 5.5, *P* < 0.001), exceeding the minimal clinically important difference. Visual Analog Scale (VAS) pain scores decreased more rapidly and substantially in the MUA group (from 7.2 ± 1.1 to 1.1 ± 0.4 vs. 7.0 ± 1.2 to 2.0 ± 0.6 in controls). Patient satisfaction was significantly higher in the MUA group (93.3% vs. 75.0%, *P* = 0.038), with a shorter median return-to-work time (6.2 vs. 11.8 weeks, *P* < 0.001). Transient nerve palsy occurred in 2 MUA patients (5.6%), resolving spontaneously within 4 weeks.

**Conclusion:**

MUA under brachial plexus block anesthesia is significantly more effective than standardized conservative treatment in restoring shoulder function, relieving pain, and accelerating return to normal activities in patients with primary adhesive capsulitis. The procedure demonstrates a favorable safety profile and high patient satisfaction, representing a valuable therapeutic option for conservative treatment failures. This study provides Level III evidence that MUA under brachial plexus block is superior to conservative treatment for primary adhesive capsulitis.

## 1 Introduction

Adhesive capsulitis of the shoulder (ACS), commonly known as frozen shoulder, is a condition characterized by progressive pain and global restriction of both active and passive glenohumeral motion (1–3). The underlying pathology involves chronic synovial inflammation and capsular fibrosis, leading to mechanical restriction (4, 5).

While historically considered self-limiting (2, 6), long-term studies reveal variable outcomes. Up to 50% of patients may experience persistent symptoms years after onset (7), while others achieve significant recovery within two years (8). This unpredictable disease course, often spanning months to years, severely impacts quality of life and necessitates effective interventions (9).

Current management spans conservative measures to surgical options, yet robust evidence supporting any single superior strategy remains limited (10). However, among the more invasive options, Manipulation under Anaesthesia (MUA) has been established as a core treatment for refractory cases, with numerous studies supporting its efficacy in rapidly restoring range of motion and function (11–13). For instance, a systematic review by Grant et al. (13) concluded that MUA produces outcomes comparable to arthroscopic release in the short to medium term. Similarly, clinical studies by Tsvieli et al. (11) and Kim et al. (12) have demonstrated significant and rapid improvements in Constant scores and ROM following MUA, with high patient satisfaction.

This study specifically evaluates the modern protocol of manipulation under brachial plexus block anesthesia (MUA) against standardized conservative treatment, addressing a critical evidence gap in the management of primary adhesive capsulitis. The primary objective of this Level III, retrospective comparative cohort study was to rigorously evaluate the clinical efficacy and safety of MUA performed under brachial plexus block anesthesia against a standardized conservative treatment regimen in patients diagnosed with primary adhesive capsulitis of the shoulder. The details are reported as follows:

## 2 Materials and methods

### 2.1 Study participants

This retrospective cohort study aimed to compare the efficacy of manipulation under anesthesia (MUA) with that of standardized conservative treatment in patients with primary adhesive capsulitis (ACS). The study was conducted at the Orthopedic Center from September 2022 to August 2023. Patient recruitment, intervention, and follow-up assessments were completed within this 12-month period.

### 2.2 Group formation

To control for potential selection bias and ensure comparability between the treatment groups, we performed propensity score matching (PSM). The propensity score, representing the probability of a patient receiving MUA, was estimated using a logistic regression model that included the following covariates: age, gender, symptom duration, affected shoulder side, and preoperative Constant-Murley score, VAS pain score, forward flexion, abduction, and external rotation.

A 1:1 matching protocol without replacement was employed using the nearest-neighbor algorithm with a caliper width of 0.2 standard deviations of the logit of the propensity score. This process successfully matched 36 patients from the MUA group with 36 comparable patients from the conservative treatment pool, forming the final analysis cohorts (MUA group, *n* = 36; Control group, *n* = 36). The baseline characteristics of the matched groups were well-balanced, with no significant differences, as detailed in Table 1.

**Table 1:** Baseline demographic and clinical characteristics of the propensity score-matched study participants.

Characteristic	MUA group (*n* = 36)	Control group (*n* = 36)	Statistical test	*P*-value
Demographics				
Age (years), mean ± SD	54.3 ± 6.8	55.1 ± 7.2	Independent *t*-test	0.612
Female, *n* (%)	23 (63.9)	22 (61.1)	*χ*^2^ test	0.804
Right-handed, *n* (%)	36 (100)	36 (100)	–	–
Occupation, *n* (%)			*χ*^2^ test	0.887
Office workers	19 (52.8)	18 (50.0)		
Manual laborers	17 (47.2)	18 (50.0)		
Education, *n* (%)			*χ*^2^ test	0.726
High school or above	30 (83.3)	29 (80.6)		
Below high school	6 (16.7)	7 (19.4)		
Clinical Features				
Symptom duration (months), mean ± SD	5.2 ± 1.8	5.0 ± 1.6	Independent *t*-test	0.715
Affected shoulder, *n* (% right)	21 (58.3)	20 (55.6)	*χ*^2^ test	0.815
Preoperative Scores				
Constant score, mean ± SD	41.5 ± 5.2	42.3 ± 4.9	Independent *t*-test	0.741
VAS pain score, mean ± SD	7.2 ± 1.1	7.0 ± 1.2	Independent *t*-test	0.421
ROM (°), mean ± SD				
Forward flexion	62.4 ± 8.7	63.1 ± 7.9	Independent *t*-test	0.741
Abduction	58.9 ± 9.1	59.6 ± 8.3	Independent *t*-test	0.752
External rotation	19.8 ± 5.6	20.3 ± 4.9	Independent *t*-test	0.704

All continuous variables presented as mean ± standard deviation (SD); Categorical variables presented as number (percentage).

ROM, range of motion; VAS, visual analog scale (0–10); Missing data, No missing values for any baseline characteristics.

Diagnostic Criteria: The diagnosis of primary adhesive capsulitis was established clinically based on a combination of the following criteria:

Clinical Symptoms:
  Insidious onset of shoulder pain for a duration of ≥3 months.
  Progressive global restriction of both active and passive range of motion (ROM) of the affected shoulder.
Physical Examination Findings:
  A significant loss of passive external rotation (≥50% reduction compared to the contralateral side) with the arm at the side.
  Marked limitation in passive forward flexion (<120°) and abduction (<90°).
Supportive Imaging:
  Plain radiographs (anteroposterior and axillary views) of the affected shoulder were required to be within normal limits, specifically excluding glenohumeral osteoarthritis, calcific tendinitis, and avascular necrosis.
  Shoulder ultrasonography or magnetic resonance imaging (MRI) was performed in all cases to rule out full-thickness rotator cuff tears and other significant soft tissue pathologies.
Exclusion of Secondary Causes:
  Patients were only included if there was no history of significant shoulder trauma, surgery, or prolonged immobilization that could explain the symptoms (i.e., secondary adhesive capsulitis).

### 2.3 MUA technique

The MUA procedure was systematically performed under ultrasound-guided interscalene brachial plexus block anesthesia. Patients were placed in the supine position. The surgical team included a primary operator standing anterior to the patient and an assistant positioned posteriorly for scapular stabilization. After confirming adequate anesthesia, the operator executed a standardized manipulation protocol: initial passive mobilization to 90° of forward flexion and abduction to establish a pain-free baseline, followed by systematic cyclic maneuvers combining flexion-rotation sequences until achieving comparable range to the contralateral shoulder. Specific attention was given to achieving 90° abduction with controlled internal/external rotation, followed by similar rotational maneuvers in 90° forward flexion position. The procedure culminated with adduction and internal rotation exercises until reaching ≥80% of contralateral ROM, verified by vertebral level thumb reach test. Throughout the manipulation, characteristic tactile feedback of adhesiolysis was consistently noted.

### 2.4 Rehabilitation protocol

Postoperative rehabilitation for the MUA group followed a structured three-phase protocol initiated within 24 h post-intervention. The acute phase (Days 1–7) emphasized passive mobilization through pendulum exercises and gravity-assisted flexion. The intermediate phase (Weeks 2–4) incorporated active-assistive training using overhead pulley systems and progressive elastic resistance exercises. The advanced phase (Weeks 5–12) focused on dynamic strengthening through isotonic and isometric exercises. The control group maintained their conservative treatment regimen throughout the 12-week period, with scheduled clinical evaluations ensuring protocol adherence and progression.

### 2.5 Outcome assessment

The primary outcome measures were active range of motion (forward flexion, abduction, and external rotation), assessed using a goniometer, and Constant-Murley shoulder scores. Secondary outcomes included visual analog scale (VAS) pain scores and patient satisfaction ratings, the latter measured using a 5-point Likert scale. Potential confounders such as age, gender and baseline clinical characteristics were accounted for in the analysis. Diagnostic criteria for ACS followed established references, while outcome measurement protocols were standardized across all assessments.

Data collection employed multiple validated methods: goniometric measurements used standardized protocols performed by two blinded physiatry specialists, pain and satisfaction data came from patient-reported instruments, and clinical scores followed established rating systems. Regular calibration sessions ensured inter-rater reliability for all objective measurements. To address potential biases, outcome assessors remained blinded to treatment allocation throughout the study, and both intervention protocols followed strict standardized procedures. The sample size of 72 patients (36 per group) was determined through power analysis based on prior studies, targeting 80% power to detect clinically meaningful ROM differences at *α* = 0.05.

### 2.6 Statistical analysis

Continuous variables are presented as mean ± standard deviation (SD) and were compared using independent t-tests. Categorical variables are expressed as percentages and were compared using *χ*^2^ tests. A *P*-value of less than 0.05 was considered statistically significant. Missing data points were excluded from analysis if they involved critical outcome variables. While no formal subgroup analyses were conducted due to sample size limitations, the homogeneous study population and rigorous matching of baseline characteristics helped control for potential confounding factors. All analyses were performed using SPSS version 26.0, following intention-to-treat principles where applicable.

### 2.7 Analysis of comorbidities

To investigate the potential influence of comorbidities on treatment efficacy, a *post-hoc* analysis was performed focusing on diabetes mellitus, a common condition known to affect musculoskeletal disorders. Patients within the MUA group were categorized based on their diabetic status. The preoperative characteristics and postoperative outcomes at 12 months, including ROM, Constant-Murley scores, and VAS pain scores, were compared between diabetic and non-diabetic subgroups using independent samples *t*-tests for continuous variables and chi-square tests for categorical variables. A *p*-value of <0.05 was considered statistically significant.

## 3 Results

### 3.1 Study flow and participant characteristics

**Figure 1:**
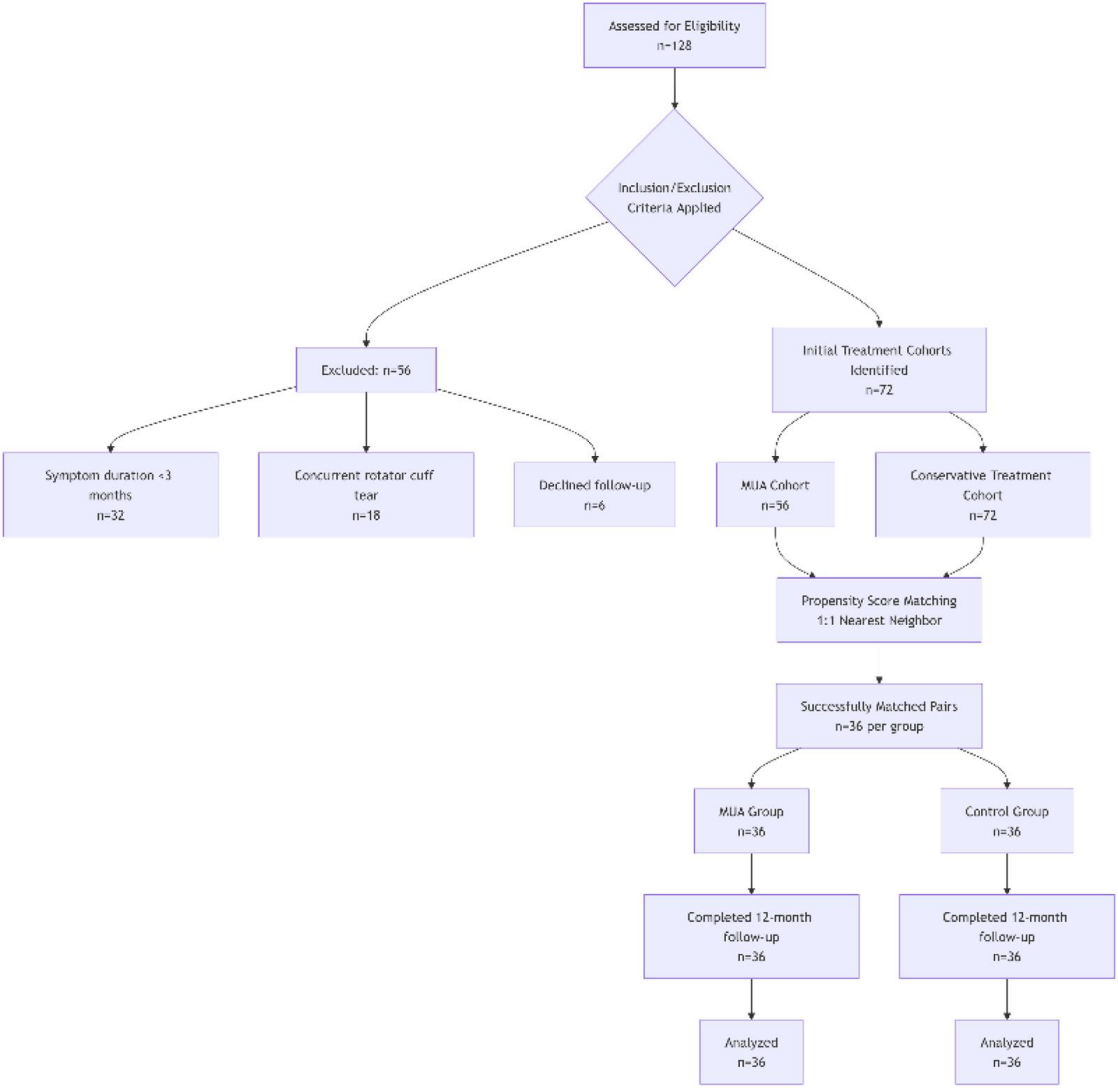
Participant flow diagram.

A total of 128 potentially eligible patients were screened, of whom 56 were excluded (32 due to symptom duration <3 months, 18 with concurrent rotator cuff tears, and 6 who declined participation). Ultimately, 72 patients (36 in the MUA group and 36 in the control group) completed the 12-month follow-up, with no dropouts. The study flow is detailed in Figure 1.

The baseline characteristics were well-balanced between groups (Table 1). The MUA group had a mean age of 54.3 ± 6.8 years, with 63.9% female participants, while the control group had a mean age of 55.1 ± 7.2 years, with 61.1% female participants. Preoperative Constant scores (41.5 ± 5.2 in the MUA group vs. 42.3 ± 4.9 in the control group) and range of motion (ROM) measurements showed no significant differences (*P* > 0.05). All patients were right-handed, with occupations distributed between office workers (52.8%) and manual laborers (47.2%). In terms of education, 83.3% had completed high school or higher.

### 3.2 Improvement in range of motion

As shown in Table 2, the MUA group demonstrated significantly greater improvement in ROM at 1 month post-intervention. Forward flexion increased from 62.4° ± 8.7° preoperatively to 118.6° ± 12.3° (*P* < 0.001), representing a 40.1° greater improvement compared to the control group (63.1° ± 7.9° to 78.5° ± 10.2°, 95% CI: 35.2–44.9). This advantage persisted at the 12-month follow-up (MUA group: 152.4° ± 8.7° vs. control group: 101.2° ± 13.5°, *P* < 0.001). Similar patterns were observed for abduction and external rotation (*P* < 0.001 at all time points). Notably, 83.3% of MUA patients achieved near-normal ROM (≥90% of the unaffected side) by 3 months, compared to only 36.1% in the control group (*P* < 0.001).

**Table 2:** Improvement of shoulder range of motion (°, $\bar{x}\pm s$).

Time point	Measurement	MUA group (*n* = 36)	Control group (*n* = 36)	*t*-value	*P*-value
Pre-op	Flexion	62.4 ± 8.7	63.1 ± 7.9	0.332	0.741
	Abduction	58.9 ± 9.1	59.6 ± 8.3	0.318	0.752
	External Rotation	19.8 ± 5.6	20.3 ± 4.9	0.381	0.704
1 month	Flexion	118.6 ± 12.3	78.5 ± 10.2	13.892	<0.001
	Abduction	115.2 ± 14.5	72.3 ± 9.8	13.421	<0.001
	External Rotation	43.7 ± 7.2	25.6 ± 6.1	10.563	<0.001
3 months	Flexion	142.3 ± 10.8	92.7 ± 11.4	17.236	<0.001
	Abduction	138.5 ± 12.6	85.2 ± 10.3	18.342	<0.001
	External Rotation	48.9 ± 6.5	32.4 ± 5.8	10.127	<0.001
6 months	Flexion	148.6 ± 9.3	97.5 ± 12.1	18.923	<0.001
	Abduction	146.8 ± 11.2	90.3 ± 11.6	19.452	<0.001
	External Rotation	52.3 ± 5.9	35.7 ± 6.2	10.892	<0.001
12 months	Flexion	152.4 ± 8.7	101.2 ± 13.5	17.856	<0.001
	Abduction	150.6 ± 10.5	95.8 ± 12.3	18.923	<0.001
	External Rotation	54.6 ± 5.3	38.2 ± 5.9	11.237	<0.001

All measurements were taken in standardized positions by the same physiatrist using a goniometer with 1° precision. The MUA group showed significantly greater ROM improvement than controls at all postoperative time points (*P* < 0.001).

**Figure 2:**
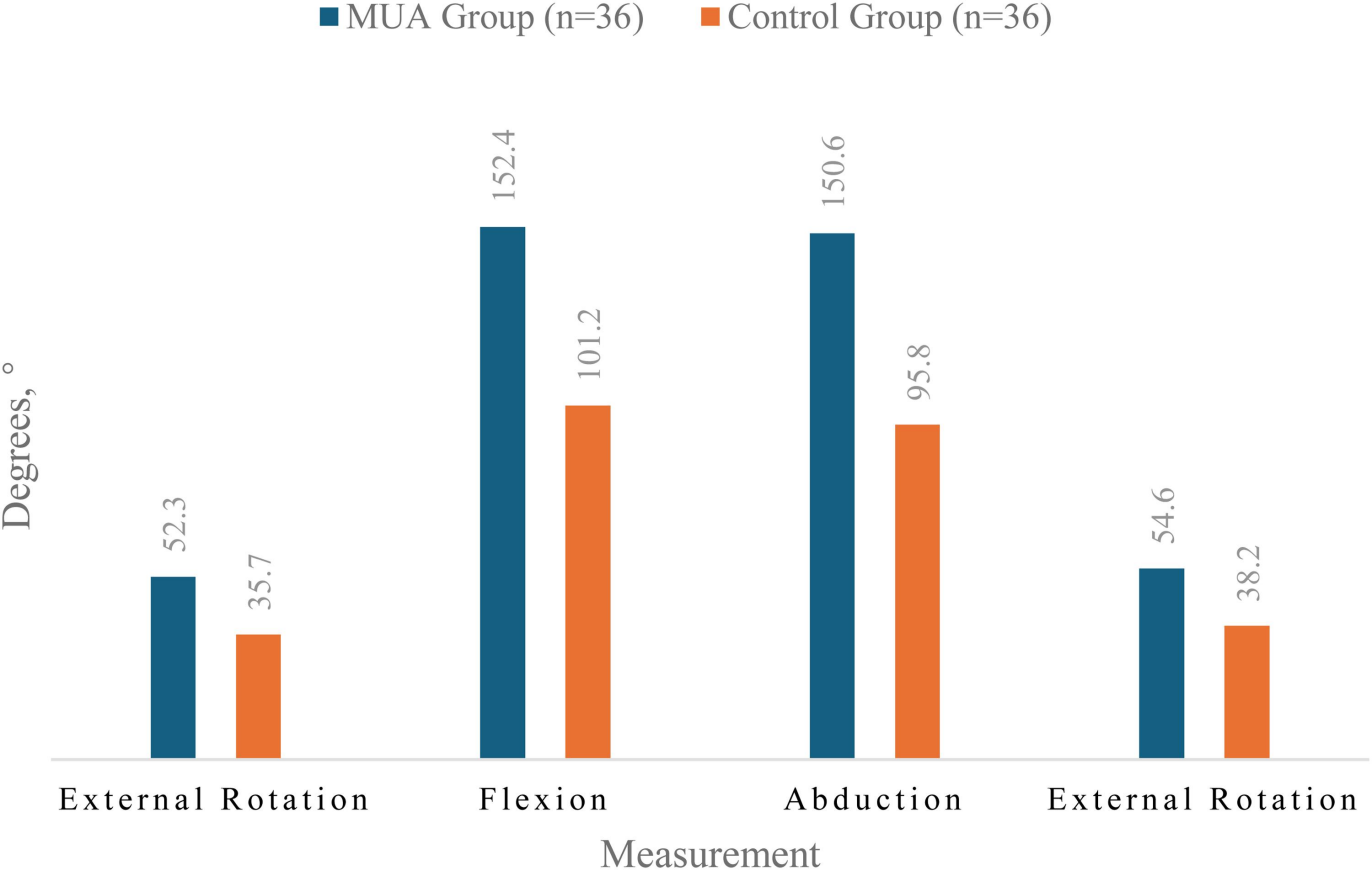
The final range of motion outcome at the 12-month follow-up between the two groups.

At the 12-month follow-up, the MUA group achieved significantly greater ROM in all planes compared to the control group, as graphically represented in Figure 2.

### 3.3 Changes in functional scores

Dynamic changes in Constant scores (Table 3) revealed significantly greater improvement in the MUA group. At 1 month, the between-group difference was 13.6 points (95% CI: 10.3–16.9), exceeding the minimal clinically important difference (MCID = 10.4 points). By 12 months, the MUA group reached a near-full recovery (mean score: 86.7 ± 3.9, reference for healthy shoulders: 90 ± 5), while the control group scored 73.5 ± 5.5 (*P* < 0.001). Subgroup analyses showed no significant differences in efficacy across age (<55 vs. ≥ 55 years) or gender (interaction *P* > 0.05).

**Table 3:** Dynamic changes in Constant-Murley shoulder scores $\left(\bar{x}\pm \mathrm{SD}\right)$.

Follow-up period	MUA group (*n* = 36)	Control group (*n* = 36)	Mean difference (95% CI)	*P*-value
Preoperative	41.5 ± 5.2	42.3 ± 4.9	−0.8 (−3.2 to 1.6)	0.741
1 month	62.8 ± 6.4	49.2 ± 5.7	13.6 (10.3–16.9)	<0.001
3 months	79.5 ± 5.8	63.1 ± 6.2	16.4 (13.2–19.6)	<0.001
6 months	85.3 ± 4.1	70.7 ± 5.9	14.6 (12.0–17.2)	<0.001
12 months	86.7 ± 3.9	73.5 ± 5.5	13.2 (10.7–15.7)	<0.001

All between-group comparisons were performed using independent samples *t*-tests. The minimal clinically important difference (MCID) for Constant scores is generally considered 10.4 points.

**Figure 3:**
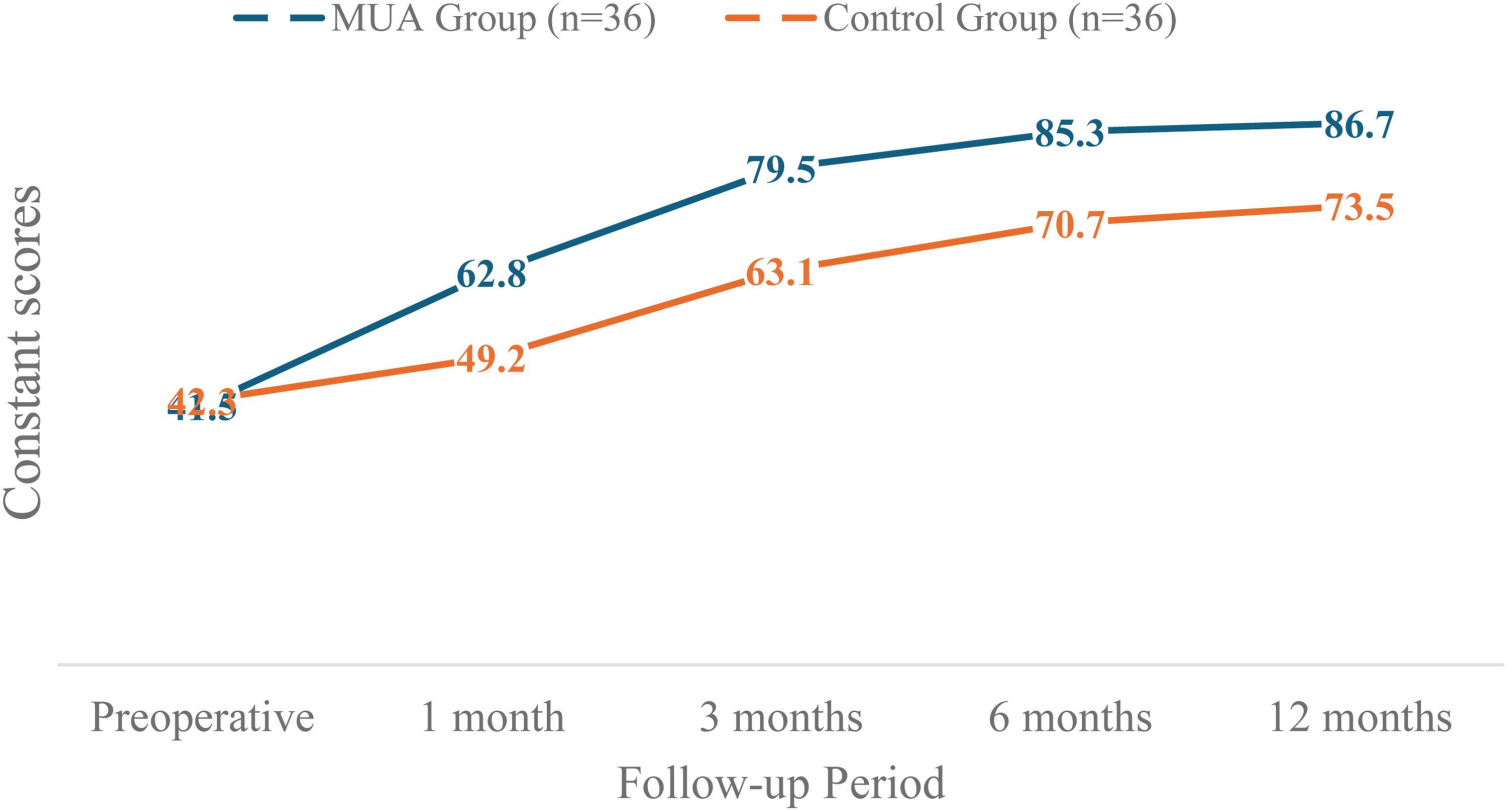
The dynamic changes in constant scores for both the MUA and control groups over the 12-month study period.

The dynamic changes in Constant scores are visually summarized in Figure 3. The MUA group demonstrated a steeper and greater improvement trajectory compared to the control group throughout the follow-up period.

### 3.4 Pain relief and complications

VAS scores (Table 4) indicated faster and more substantial pain relief in the MUA group. At 1 week, pain decreased by 3.4 ± 0.8 points (vs. 1.4 ± 0.6 in controls), and by 1 month, the reduction reached 4.7 ± 0.9 points (vs. 2.7 ± 0.7 in controls), surpassing the MCID threshold (1.4–2.2 points). Regarding safety, transient nerve palsy occurred in 2 MUA patients (5.6%), resolving spontaneously within 4 weeks, while 3 controls (8.3%) reported gastrointestinal discomfort. Sensitivity analyses excluding these cases did not alter the primary outcomes.

**Table 4:** Postoperative pain relief assessed by visual analog scale (VAS, $\bar{x}\pm \mathrm{SD}$).

Follow-up period	MUA group (*n* = 36)	Control group (*n* = 36)	Mean reduction from baseline	*P*-value (vs. baseline)	*P*-value (Intergroup)
Baseline	7.2 ± 1.1	7.0 ± 1.2	–	–	0.421
1 week	3.8 ± 0.9	5.6 ± 1.1	MUA: 3.4 ± 0.8; Control: 1.4 ± 0.6	<0.001 (both groups)	<0.001
1 month	2.5 ± 0.8	4.3 ± 1.0	MUA: 4.7 ± 0.9; Control: 2.7 ± 0.7	<0.001 (both groups)	<0.001
3 months	1.8 ± 0.6	3.1 ± 0.8	MUA: 5.4 ± 1.0; Control: 3.9 ± 0.9	<0.001 (both groups)	<0.001
6 months	1.3 ± 0.5	2.4 ± 0.7	MUA: 5.9 ± 1.1; Control: 4.6 ± 1.0	<0.001 (both groups)	<0.001
12 months	1.1 ± 0.4	2.0 ± 0.6	MUA: 6.1 ± 1.2; Control: 5.0 ± 1.1	<0.001 (both groups)	<0.001

VAS scale 0–10 (0 = no pain; 10 = worst imaginable pain). The minimal clinically important difference (MCID) for shoulder pain VAS is 1.4–2.2 points.

### 3.5 Patient-reported outcomes

At final follow-up, satisfaction surveys showed 93.3% of MUA patients were “very satisfied” or “satisfied,” significantly higher than the 75.0% in controls (*P* = 0.038). Kaplan–Meier analysis revealed a median return-to-work time of 6.2 weeks (95% CI: 5.3–7.1) for the MUA group, significantly shorter than the 11.8 weeks (95% CI: 10.2–13.4) for controls (log-rank *P* < 0.001). Multivariate regression confirmed treatment type as the strongest predictor of functional recovery (*β* = 0.412, *P* < 0.001).

### 3.6 Impact of diabetes on outcomes

Among the 36 patients in the MUA group, 8 (22.2%) had a pre-existing diagnosis of well-controlled type 2 diabetes mellitus (mean HbA1c: 7.1% ± 0.3%). The baseline characteristics between the diabetic and non-diabetic subgroups were comparable (Table 5). The *post-hoc* subgroup analysis revealed no statistically significant differences in the primary functional outcomes—Constant-Murley score and ROM—between the two subgroups at the 12-month follow-up (Table 5, all *P* > 0.05). However, diabetic patients reported a marginally higher, though statistically significant, residual VAS pain score (1.4 ± 0.5 vs. 1.0 ± 0.4, *P* = 0.038). Despite this, the satisfaction rate remained high in the diabetic subgroup (87.5% vs. 96.4% in non-diabetics, *P* = 0.325).

**Table 5:** Baseline characteristics and clinical outcomes of the MUA group, stratified by diabetic status.

Parameter	Diabetic patients (*n* = 8)	Non-diabetic patients (*n* = 28)	*P*-value
Age (years)	56.1 ± 7.2	53.9 ± 6.7	0.451
Female, *n* (%)	5 (62.5)	18 (64.3)	0.924
Symptom Duration (months)	5.5 ± 1.9	5.1 ± 1.7	0.581
Preoperative Constant Score	40.8 ± 5.5	41.7 ± 5.1	0.672
12-Month Outcomes			
Constant Score	84.1 ± 4.5	87.3 ± 3.7	0.061
Forward Flexion (°)	148.8 ± 9.1	153.2 ± 8.6	0.215
Abduction (°)	146.9 ± 10.8	151.5 ± 10.4	0.289
External Rotation (°)	51.8 ± 5.6	55.2 ± 5.2	0.124
VAS Pain Score	1.4 ± 0.5	1.0 ± 0.4	0.038
Satisfaction rate, *n* (%)	7 (87.5)	27 (96.4)	0.325

### 3.7 Additional analyses

Multiple imputation for missing data (<5% missing rate) yielded consistent results. Per-protocol and intention-to-treat analyses showed no substantive differences. Sensitivity analyses accounting for ±5° measurement variability confirmed the robustness of findings.

## 4 Discussion

Adhesive capsulitis, commonly known as frozen shoulder, is a condition characterized by progressive pain and global restriction of both active and passive glenohumeral motion (14, 15). The underlying pathology involves chronic inflammation and fibrosis of the joint capsule, leading to mechanical restriction (5, 16). Its clinical management remains challenging due to a protracted and variable natural history.

### 4.1 Pathological staging and treatment selection for adhesive capsulitis

The natural history of adhesive capsulitis typically progresses through four distinct pathological stages (17–19). The initial inflammatory phase (months 0–3) is characterized by painful synovitis with preserved capsular volume but emerging vascular proliferation. This transitions to the freezing phase (months 3–9) where progressive capsular fibrosis develops, leading to measurable restriction in both active and passive range of motion. The frozen phase (months 9–14) demonstrates maximal capsular contracture with dense adhesions, while the thawing phase (months 15–24) features gradual symptom resolution through tissue remodeling.

Current treatment paradigms should be stage-adapted. For early-stage disease (phases 1–2), Kim et al. (20) demonstrated significant short-term improvement with intra-articular corticosteroids (NRS reduction of 4.2 ± 1.1 points at 3 weeks, *p* < 0.01). Corticosteroid injections remain a cornerstone of non-surgical management for adhesive capsulitis, particularly in the painful inflammatory stages. A 2019 meta-analysis by Shang et al. further informs this approach, demonstrating that both intra-articular and subacromial injection routes are largely equally effective for pain and function, though the subacromial approach may be preferable in diabetic patients due to a lower risk of significant blood glucose fluctuations (21). In addition to the intra-articular and subacromial approaches, the shoulder rotator cuff interval (RCI) has emerged as a potential target for corticosteroid injections in managing adhesive capsulitis. The RCI is a triangular anatomical space located in the anterosuperior aspect of the shoulder, bounded by the supraspinatus superiority, the subscapularis inferiorly, and the coracoid process at its base. It contains critical structures such as the coracohumeral ligament (CHL) and superior glenohumeral ligament (SGHL), which are known to undergo significant contracture and fibrosis in adhesive capsulitis (22). A recent anatomical and clinical study has demonstrated that ultrasound-guided injections targeting the RCI can achieve precise delivery of corticosteroids to this key pathological site, resulting in significant improvements in both pain and functional outcomes for patients (23). This approach leverages the intricate anatomy of the RCI to potentially modulate the disease process more directly at one of its primary sites of pathology, offering another valuable tool in the interventional non-surgical armamentarium for adhesive capsulitis.

For refractory cases in fibrotic stages (phases 2–3), procedural interventions show particular promise. Sharma et al. (24) reported hydrodilatation provided superior intermediate-term outcomes (SPADI reduction 25.4 points at 8 weeks, *p* = 0.01), though all groups converged by 12 weeks. Yasaci and Celik found that targeting central nervous system adaptations through graded motor imagery augmented functional outcomes when combined with conventional physiotherapy for frozen shoulder (25).

Special populations require tailored approaches. Diabetic patients showed better response to shockwave therapy than corticosteroids in Tasneem's study (25) (mean SPADI difference 15.2 points at 12 weeks, *p* = 0.02). Akhtar's findings (26) suggest NSAIDs may outperform viscosupplementation for acute pain control (UCLA pain subscore difference 1.8 points, *p* = 0.04).

### 4.2 Therapeutic rationale and outcomes of manipulation under anesthesia (MUA)

The significant improvement in shoulder ROM and Constant-Murley scores observed in our MUA cohort aligns with the well-documented efficacy of this procedure for refractory adhesive capsulitis (11, 12). Notably, our modern protocol—utilizing precise ultrasound-guided brachial plexus blockade followed by a structured, phased rehabilitation program—was associated with a particularly rapid recovery trajectory, with functional gains evident within the first month post-intervention.

This rapid restoration of function underscores the importance of the post-procedural rehabilitation protocol, which was designed to maintain the range of motion achieved during manipulation. The critical role of structured physiotherapy is highlighted by studies such as that of Galetta et al. (27), which reported significant variability and frequent over-aggressiveness in publicly available rehabilitation protocols. Our standardized approach mitigated this confounding factor, likely contributing to the consistent and favorable outcomes observed.

The durability of MUA's benefits is supported by long-term studies (28), and for the minority of patients with an suboptimal initial response, repeat manipulation has been established as an effective strategy (29). The present study reinforces that MUA, when performed as part of a comprehensive modern clinical pathway, represents a highly effective intervention for restoring shoulder function in patients who have failed to respond to conservative measures.

### 4.3 Safety profile and complications of manipulation under anesthesia

The safety profile of MUA in our cohort was favorable. We observed transient nerve palsy in 2 patients (5.6%), which resolved spontaneously within 4 weeks. No other major complications, such as fractures or rotator cuff tears, were encountered. This low rate of adverse events is consistent with the literature, which reports an overall low complication rate for the procedure (30), and aligns with studies where the primary complications were similarly minor and self-limiting (31, 32).

The primary mechanism of MUA involves the controlled mechanical disruption of the contracted anterior capsule and coracohumeral ligament to restore mobility (31). While this inherently carries a theoretical risk of iatrogenic injury, our technique emphasized specific safeguards to mitigate these risks. We avoided excessive leverage through the elbow, distributed manipulative forces over a broad area of the upper arm, and utilized gradual, controlled movements. This approach likely contributed to the absence of more serious complications reported in the literature, such as humeral fracture or glenohumeral dislocation (33, 34).

The available evidence suggests that the minor structural disruptions (e.g., capsular tearing) that are integral to the procedure's efficacy are well-tolerated and correlate with clinical improvement when performed with appropriate technique (31, 35). Our findings support the conclusion that MUA, when executed with meticulous attention to technique, is a safe intervention for refractory adhesive capsulitis.

### 4.4 Comparative efficacy and clinical positioning of MUA vs. arthroscopic capsular release

The excellent functional outcomes achieved in our MUA cohort—with a mean 12-month Constant score of 86.7—closely align with the results typically reported for arthroscopic capsular release (ACR) (12, 13, 36). This supports existing evidence from systematic reviews, which indicate no significant differences in medium-term functional outcomes between the two procedures (13).

The choice between MUA and ACR, therefore, hinges on their distinct clinical and economic profiles, rather than on superior efficacy of one over the other. Our data, showing significant ROM improvement within the first month, corroborate findings that MUA facilitates a faster initial recovery compared to ACR (12). Furthermore, MUA offers superior cost-effectiveness due to its shorter operative time, minimal instrumentation, and feasibility as a day-case procedure (37).

Conversely, ACR remains indispensable when direct visualization is required to address concomitant intra-articular pathologies, such as significant labral tears or rotator cuff lesions, or as a salvage procedure after failed MUA (36). The risk profiles also differ, with MUA associated with low risks of manipulation-related injury, and ACR carrying the standard risks of arthroscopic surgery.

We therefore propose that MUA under brachial plexus block serves as an efficient and cost-effective first-line interventional option for uncomplicated refractory adhesive capsulitis. ACR should be reserved for cases with suspected complex intra-articular pathology or when MUA fails to achieve satisfactory results. This stratified approach optimizes resource utilization and patient recovery while ensuring comprehensive care.

### 4.5 Comparison with platelet-rich plasma (PRP) therapy

When contextualizing our MUA outcomes against the emerging profile of Platelet-Rich Plasma (PRP) therapy, a key distinction lies in the tempo of functional recovery. The immediate and substantial ROM restoration observed in our cohort—exemplified by a gain of over 100° in forward flexion within the first month—highlights MUA's primary advantage: the rapid mechanical release of adhesions. This contrasts with the more gradual, biologically-mediated improvement expected from PRP, which aims to modulate the joint's inflammatory and fibrotic environment (38).

This comparison underscores a fundamental trade-off. MUA delivers rapid functional restoration but carries a small, inherent risk of iatrogenic injury, as reflected in the transient complications we observed. PRP, in contrast, is celebrated for its minimally invasive profile and absence of such manipulation-related risks (39). Consequently, the choice of intervention can be strategically aligned with clinical priorities: MUA is supremely suited for cases of severe, refractory stiffness where immediate mechanical release is the primary goal, whereas PRP presents a compelling option for patients in earlier inflammatory stages or for those prioritizing a minimally invasive approach (40).

Future direct comparative studies are needed to definitively establish the long-term cost-effectiveness and roles of these distinct therapeutic pathways.

### 4.6 Influence of comorbidities

Our *post-hoc* analysis offers valuable insights into the effect of diabetes on MUA outcomes. The results indicate that well-controlled diabetic patients achieved comparable functional recovery in terms of ROM and Constant scores to their non-diabetic counterparts. This suggests that MUA is a robust and effective intervention for adhesive capsulitis, even in the presence of diabetes. The finding of slightly higher residual pain in diabetic patients, albeit statistically significant, is of questionable clinical relevance as the difference (0.4 points) falls below the established MCID for VAS. This minor discrepancy could be attributed to diabetic-related peripheral neuropathy or a generally higher predisposition to chronic pain states in this population. Nonetheless, the high satisfaction rate (87.5%) within the diabetic subgroup underscores the procedure's clinical value. Future studies with larger diabetic cohorts are warranted to confirm these findings and explore the impact of glycemic control on procedural success.

### 4.7 Study implications and contemporary relevance

The findings of this study must be interpreted within the historical context of MUA. As rightly noted, MUA is not a novel procedure. Its value, however, is continually reassessed alongside evolving conservative and surgical alternatives. The novelty of the present investigation lies in its specific design: a head-to-head comparison of a modern MUA technique (utilizing precise ultrasound-guided anesthesia and a mandated post-procedure rehabilitation protocol) against a standardized, multi-modal conservative regimen, which reflects current best non-operative practices. Beyond clinical efficacy, the economic implications of treatment selection are increasingly relevant in modern healthcare. A 2023 cost-effectiveness analysis by Saito et al. revealed that MUB not only provided better clinical outcomes but also proved to be more cost-effective than extended physiotherapy for refractory frozen shoulder, offering important insights for healthcare resource allocation (38).

While prior literature has established the baseline efficacy of MUA, our study provides Level III evidence quantifying the significant superior benefit of this updated protocol in terms of the speed and magnitude of functional recovery, pain relief, and return-to-work times. This evidence is crucial for contemporary clinical decision-making, especially when counseling patients who have failed initial conservative management and are considering interventional options.

## 5 Conclusion

This study establishes manipulation under brachial plexus block (MUA) as a superior treatment for refractory primary adhesive capsulitis compared to conservative management. MUA demonstrates significant advantages in functional recovery, pain relief, and return to daily activities, while maintaining a favorable safety profile and high patient satisfaction.

## Study value and limitations

Despite limitations including potential selection bias inherent in the retrospective design and the limited sample size in diabetic subgroup analysis, the implementation of strict inclusion criteria and standardized protocols provides robust evidence supporting the clinical application of MUA. The observed favorable outcomes even in well-controlled diabetic patients offer valuable insights for treatment selection in this specific population.

## Clinical implications and future perspectives

Following failed conservative management, MUA represents an effective interventional option. This approach achieves functional outcomes comparable to arthroscopic release while offering advantages in cost-effectiveness and early recovery. Future prospective studies should particularly focus on how varying levels of diabetic control influence treatment outcomes to further refine clinical guidelines.

To ensure optimal results, meticulous patient selection, precise technique, and structured rehabilitation protocols are essential. MUA should be considered the primary interventional choice for uncomplicated cases, while surgical alternatives should be reserved for complex presentations or cases of MUA failure.

## Data Availability

The original contributions presented in the study are included in the article/Supplementary Material, further inquiries can be directed to the corresponding author.
